# Effects of biopolymers, cork, and *Rhizobium tropici*-derived extracellular polymeric substances on soil microbial communities

**DOI:** 10.3389/frmbi.2025.1614472

**Published:** 2025-08-15

**Authors:** Alexis K. Craft, Sowndarya Karapareddy, Varsha C. Anche, Madhusudhana R. Janga, Obaloluwa Soyinka, Sravan K. Sanathanam, Seloame T. Nyaku, Govind C. Sharma, Zachary Senwo, Venkateswara R. Sripathi

**Affiliations:** ^1^ College of Agricultural, Life & Natural Sciences, Alabama A&M University, Normal, AL, United States; ^2^ Institute of Genomics for Crop Abiotic Stress Tolerance, Department of Plant and Soil Science, Texas Tech University, Lubbock, TX, United States; ^3^ Department of Crop Science, College of Basic and Applied Sciences, University of Ghana, Accra, Ghana

**Keywords:** soil, amendments, time points, treatments, bacterial communities, microbial diversity

## Abstract

**Introduction:**

Soil microorganisms play a crucial role in plant development, while biopolymers, such as cork and Extracellular Polymeric Substances/Exopolysaccharides (EPS), can enhance soil health. However, these amendments may affect DNA extraction and microbial analysis, necessitating the validation of the extraction method before conducting next-generation sequencing (NGS).

**Methods:**

This study evaluated 48 soil samples from Decatur, Alabama (Silt loam) that underwent four treatments: unamended soil (soil.control), soil with cork (soil.cork), soil with EPS (soil.EPS), and soil with both cork and EPS (soil.cork.EPS). Samples were collected at four time intervals (0-, 24-, 48-, and 72-hours post-treatment), with three biological replicates for each treatment. The FastDNA Spin Kit proved the most effective among the six DNA extraction methods tested.

**Results and discussion:**

Amplicon sequencing of the 16S rRNA gene identified 62,996 amplicon sequence variants (ASVs), with 513 ASVs shared across all time points and 467 ASVs shared among the different treatments. The microbial community was primarily composed of *Actinobacteria, Proteobacteria*, and *Acidobacteria*, with *Actinobacteria* being the most abundant phylum. *Actinobacteria, Alphaproteobacteria, Bacilli*, and *Betaproteobacteria* contributed to microbial diversity at the class level. Notable families such as *Bacillaceae, Gaiellaceae, Micromonosporaceae*, and *Streptomycetaceae* showed treatment-dependent variations. Core microbiome analysis revealed *Bacillus* and *Gaiella* as the dominant genera, which play vital roles in soil ecosystem stability and nutrient cycling. These microbes contribute to carbon sequestration, nitrogen fixation, and phosphorus solubilization, improving soil fertility and plant-microbe interactions. These findings offer valuable insights into microbial dynamics in amended soils, providing information that can improve soil quality and agricultural productivity.

## 1 Introduction

Alabama’s agricultural landscape is shaped by its diverse soil types and climatic conditions, which in turn determine its crop production and ecosystems. The state has seven main types of soil: Appalachian Plateau, Piedmont Plateau, Blackland Prairie, limestone valleys and uplands, floodplains, coastal marshes, and coastal plains (Mitchell, 2018). Among these, the Bama soil (20-35% clay, 45-80% sand, and less than 30% silt) and Decatur silt loam (35-60% clay, 15-35% sand, and 4-20% silt) moderately to highly acidic and thus limiting crop cultivation (Howe and Mitchell, 2010). Microorganisms play key roles in nutrient cycling, detoxification, and regulating soil microclimates (Cheng et al., 2021; Romero et al., 2023) and maintaining microbial diversity which is an effective soil management practice. This emphasis on soil microbial health which is an important strategy in sustainable agriculture for increasing agricultural resilience against challenges such as soil acidity (Msimbira and Smith, 2020).

Organic amendments, biofertilizers, and biopolymers can effectively reduce acidity while adjusting pH levels and enhancing crop productivity by improving soil structure, fertility, and microbial activity. Soil amendments can alter microbial communities and increase their diversity, which improves soil fertility and helps convert nutrients into forms that are readily available to plants (Dincă et al., 2022). The application of biofertilizers (compost and manure) and biopolymers (cork and Extracellular Polymeric Substances/Exopolysaccharide, EPS) has proven effective in enhancing soil fertility, nutrient retention, and promoting healthier crop growth (Lucchetta et al., 2023; Rompato et al., 2024; Redmile-Gordon et al., 2020; Zhang et al., 2024). Integrating such soil amendments with cover cropping (Carrera et al., 2007) not only contributes to sustainable agriculture and crop production but also Alabama’s rich soil tapestry.

Cork, a unique natural biopolymer, rich in suberin, derived from the bark of the cork oak (*Quercus suber* L.), has been significantly overlooked as a soil amendment despite its considerable potential to improve soil structure and microbial activity (Rompato et al., 2024). Cork improves water retention capabilities and helps buffer soil acidity (Msimbira and Smith, 2020) making it particularly beneficial in regions with higher soil acidity, such as North Alabama and Tennessee Valley. In addition, cork particles contribute to improved soil porosity, facilitating root development and promoting microbial colonization, which collectively improves overall soil health (Rompato et al., 2024). The hydrophobic nature of cork also enables it to improve aeration in heavier soils, such as silt and clay, thereby supporting diverse microbial populations. Consequently, cork presents a promising amendment for fostering improved soil structure and sustainability in high-value agro-horticultural systems (Cheng et al., 2021; Rompato et al., 2024). The unique physical and biological features of cork make it a valuable component in sustainable soil management practices.

The EPS produced by *Rhizobium tropici* enhances soil health by promoting beneficial microbial interactions, improving soil structure, increasing moisture retention, and facilitating nutrient cycling, all of which are important for maintaining soil fertility and effective agricultural practices (Tisdall and Oades, 1982; Flemming and Wingender, 2010). EPS also contributes to stabilizing soil structure, rendering it more resistant to erosion (Flemming and Wingender, 2010). Microorganisms can be favorably influenced by the synergic effects of soil amendments, such as cork and EPS, which enhance biogeochemical processes that convert organic matter into forms accessible to plants. For example, a few beneficial microbes convert organic phosphates into orthophosphates, enhancing nutrient uptake, particularly in low-pH soils where phosphorus availability is limited (Cheng et al., 2021). The synergistic effect of cork and EPS biopolymers further supports the proliferation of phosphate-solubilizing bacteria, thereby improving nutrient cycling and promoting both soil fertility and microbial diversity (Costa et al., 2018; Rompato et al., 2024; Redmile-Gordon et al., 2020).

The success of 16S rRNA sequencing and analysis is affected by the quality of input DNA, which relies on effective DNA isolation methods. Cell lysis and the removal of impurities in soil are essential for isolating quality DNA (Pu et al., 2025). Soil pH and acidity impact these methods, highlighting the need for standardized protocols. The microbial diversity in the rhizospheric region is likely to improve after soils are amended with biopolymers, which in turn promote soil structure, nutrient recycling, and beneficial interactions between plants and microbes. Using biofertilizer and biopolymer amendments helps increase soil microbial diversity, which supports crop production and reduces our dependence on chemical fertilizers. Despite the known benefits, limited studies have been conducted to assess the changes in microbial communities resulting from the use of biopolymer amendments. In the present study, we aimed to identify an effective DNA isolation method for determining microbial diversity among unamended and amended (cork and EPS) soils using 16S rRNA sequencing.

## 2 Materials and methods

### 2.1 Study area and sample collection

The soil sampling procedures used in this study strictly adhered to the Alabama Cooperative Extension System protocol (Thompson et al., 2004). The surface soil (0-15 cm) was sampled via a row-wise sampling method and transported under refrigerated conditions for further processing. The soil textural analysis identified the soil as silt loam with (17.5–26.31%), silt (69.45–78%), and clay (7.5–9%). The CNS characterization revealed carbon (8.45–8.51%), nitrogen (0.21–0.22%), and sulfur (0.18–0.19%) (**Supplementary Table 1**). The two primary amendments applied to the Decatur soil were fine powdered cork (0.1 mm) and EPS (0.02%) derived from *Rhizobium tropici*. The proportions of these amendments were determined through preliminary testing (Watts et al., 2024; Metuge and Senwo, 2024). This study utilized 48 samples, categorized into four treatments: unamended soil (soil.control), soil amended with cork (60:40, soil.cork), soil amended with 10 mL of 0.02% EPS (soil.EPS), and soil amended with both cork and EPS (soil.cork.EPS). Furthermore, the study encompassed four collection time points (Day 0-0 Hours After Treatment [HAT], Day 1-24 HAT, Day 2-48 HAT, and Day 3-72 HAT) with biological triplicates for each treatment (R1, R2, and R3), resulting in 48 samples.

### 2.2 Microbial DNA isolation

The study assessed six methods for isolating DNA, which included the following: FastDNA Spin Kit for Soil (MP Biomedicals), EZNA Soil DNA Kit (Omega Biotek), DNeasy PowerMax Soil Kit (Qiagen), PowerSoil DNA Isolation Kits (MO BIO), Quick-DNA Fecal/Soil Microbe Kit (Zymo Research), and a modified Cetyl Trimethyl Ammonium Bromide (CTAB) protocol for isolating the soil DNA using unamended soil.control, and amended soils: soil.cork, soil.EPS, and soil.cork.EPS to determine an optimal approach for 16S rRNA amplicon sequencing and subsequent microbial diversity analysis.

The genomic DNA was extracted using the FastDNA Spin Kit for Soil (MP Biomedicals), according to the manufacturer’s protocol, and eluted to 50 µl. The quantity and quality of the DNA were analyzed by Qubit 1X dsDNA Broad Range Assay Kit (Maretto et al., 2022) and Gel Electrophoresis (Green and Sambrook, 2019), respectively. The gel electrophoresis showed the size and distribution of DNA fragments, providing a detailed view of DNA integrity prior to sequencing, while the Qubit assay yielded readings of high concentration (Maretto et al., 2022; Green and Sambrook, 2019).

### 2.3 Library preparation, sequencing, and bioinformatic analyses

To target the V3–V4 region of the 16S rRNA gene, bacterial amplicon sequencing (Satam et al., 2023) was conducted using the Quick-16S NGS Library Prep Kit (Zymo Research, Irvine, CA). The PCR protocol includes initial denaturation at 95°C for 3 min, followed by 25 cycles of denaturing at 95°C for 30 sec, annealing at 55°C for 30 sec, and elongation at 72°C for 30 sec, and finishing with a final extension at 72°C for 5 min. To ensure reproducibility, each sample was processed three times.

To decrease the creation of PCR chimeras during library preparation, real-time PCR monitoring was utilized. The qPCR fluorescence readings were used to quantify the PCR products and were pooled according to equal molarity. Subsequently, the pooled library was purified with the Select-a-Size DNA Clean and Concentrator (Zymo Research, Irvine, CA) and quantified using TapeStation (Agilent Technologies, Santa Clara, CA) as well as Qubit 1X dsDNA High-Sensitivity Assay Kits (Fornasier et al., 2014). For each DNA extraction and targeted library preparation, blank extraction and library preparation controls were included to ensure quality and evaluate potential contamination. In addition, ZymoBIOMICS Microbial Community DNA Standard (Zymo Research, Irvine, CA) was also incorporated as a positive control (Song et al., 2021). The Illumina MiSeq platform with a v3 reagent kit (600 cycles) was utilized to sequence 48 libraries.

Bioinformatics analyses were performed to first filter the low-quality reads (Abellan-Schneyder et al., 2021; Johnson et al., 2019). The Divisive Amplicon Denoising Algorithm 2 (DADA2) pipeline in R (v4.3.2) was employed to process the raw reads from the 16S rRNA amplicon sequencing (Callahan et al., 2016). Data matrix construction and identification of variations in the microbiome of samples under study were performed by Phyloseq (v1.46.0). The DADA2 pipeline includes quality filtering, trimming, and truncation of forward reads at position 300 and reverse reads at position 250; de-replication to eliminate redundancy and find Amplicon Sequence Variants (ASVs); sequence table construction, chimeras removal using the command “removeBimeraDenovo”, taxonomy assignment, and phylogenetic tree construction. The taxonomy was assigned by a naive Bayesian classifier, utilizing the Ribosomal Database Project v19 training set for 16S rRNA data (Wang and Cole, 2024). The ASVs were aligned, and a phylogenetic tree was built with the DECIPHER R (Wright, 2016) and Phangorn R (Schliep, 2011) packages, respectively.

Statistical analyses and visualizations were conducted using R (v4.3.2) with the Phyloseq (v1.46.0) package. In addition, phyloseq (McMurdie and Holmes, 2013), biocStyle (Andrzej Oleś, 2017), gridExtra (Auguie and Antonov, 2017), ggplot2 (Villanueva and Chen, 2019), dada2 (Callahan et al., 2016), DECIPHER (Wright, 2016), phangorn (Schliep, 2011), magrittr (Bache et al., 2022), microbiotaProcess (Xu et al., 2023), VennDiagram (Chen and Boutros, 2011), UpsetR (Conway et al., 2017), microbiomeR (Gilmore et al., 2019), RColorBrewer (Neuwirth, 2014), dplyr (Wickham et al., 2023), microbiomeAnalyst 2.0 (Lu et al., 2023) and microbiomeUtilities (Shetty and Lahiti, 2022) packages were used. The data was imported to a Phyloseq object, and the microbiome, ggpubr, knitr, and dplyr packages were utilized to calculate alpha diversity. The weighted and unweighted Unifrac distance metrics were utilized to calculate the beta diversity (Lozupone et al., 2011). For evaluating differences in beta diversity among the time points and treatments, Permutational Multivariate Analysis of Variance (PERMANOVA) is an essential tool for soil microbiologists for calculating statistical significance of observed species composition differences (Karapareddy et al., 2025) and helps to understand how biopolymer amendments influence bacterial community structure. The Phyloseq package was used to aggregate taxonomy at the phylum, class, and family levels. The p-value ≤ 0.05 was considered statistically significant. The microbiomeAnalyst was utilized for core bacterial microbiome analysis, relying on a detection threshold of relative abundance expressed in percentage.

## 3 Results

### 3.1 Assessment of soil DNA isolation

**Figure 1:**
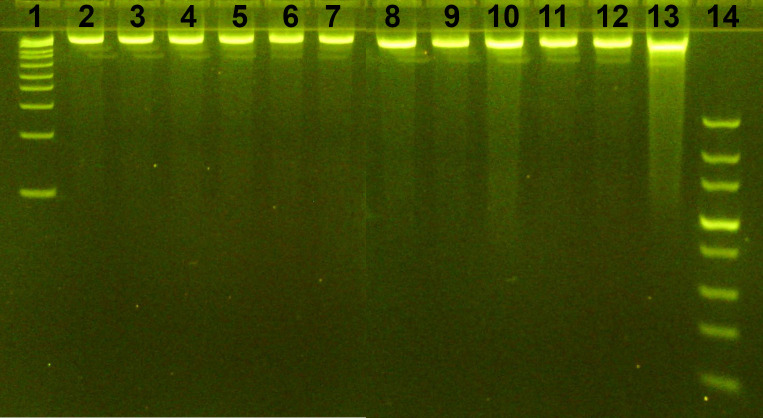
Gel (4% Agarose) electrophoresis image represents isolated DNA from different treatments of Day1 samples using FastDNA Spin Kit. 1: 1kb plus ladder; 2, 3, 4: soil.control; 5, 6, 7: soil.cork; 8, 9, 10: soil.EPS; 11, 12, 13: soil.cork.EPS; 14: Ultra-low range DNA ladder.

In this study, six different DNA extraction methods were evaluated using both amended and unamended Decatur soil samples to determine the most efficient approach for 16S rRNA amplicon sequencing and microbial diversity analysis. Among these methods, the FastDNA Soil Spin Kit demonstrated the highest efficiency, yielding high-quality DNA suitable for downstream sequencing and analysis. This method provided consistent and reproducible results, making it the preferred choice for studying soil microbial communities. DNA integrity and extraction efficiency are illustrated with a representative gel image from Day 1 (**Figure 1**), showcasing the DNA quality and quantity isolated across various methods.

### 3.2 Data processing summary

A total of 12,345,526 (12.34 million) raw paired-end reads of 16S rRNA gene sequences were obtained from 48 soil samples, which included both amended and unamended Decatur soils. After quality control and trimming with the DADA2 pipeline, 12,248,252 (12.24 million) high-quality reads were retained for further analysis. The reduction in read count resulted from the removal of low-quality bases, chimeric reads, and adapter sequences. Unique sequences were obtained after applying multiple processing steps, including trimming, dereplication, filtering of chimeric regions, and size selection (**Supplementary Table 2**). The observed variations in processed and unique read counts were primarily attributed to the elimination of duplicate sequences, chimeric artifacts, and shorter reads that did not meet the quality thresholds.

To classify taxonomically, sequences were assigned to taxa through comparison with the RDP v19 training set. The classification process revealed distinct microbial compositions across the different time points and treatments. After filtering through DADA2, sequences were grouped into unique ASVs and subsequently aligned using the DECIPHER R package. Subsequently, to organize the data, a Phyloseq object was generated to facilitate further analyses, including alpha diversity estimation, beta diversity evaluation, and taxonomic composition visualization via stacked bar plots. Additionally, core microbiome analysis and Venn diagram generation were performed to identify shared and unique microbial taxa across different time points and treatments.

### 3.3 α-diversity indices

**Figure 2:**
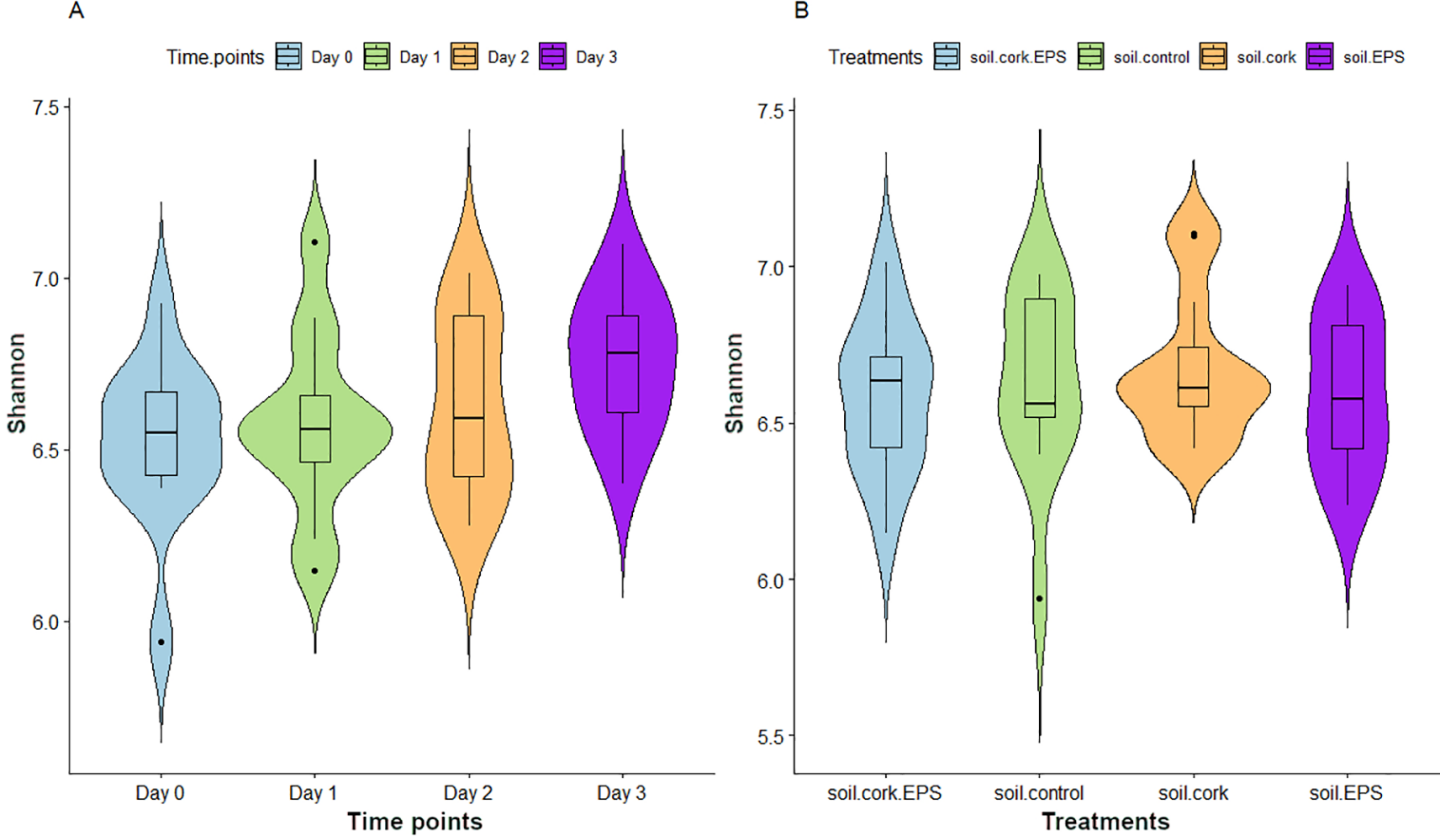
**(A, B)** Violin plots depicting the alpha diversity with bacterial species richness and evenness (Shannon Diversity Index-SDI) across different time points and treatments with statistical significance (*p* < 0.05, Kruskal–Wallis test). soil.control (soil control), soil.cork (soil+cork), soil.cork.EPS (soil+cork+EPS), soil.EPS (soil+EPS).

Bacterial community α-diversity was assessed using the Shannon Diversity Index (SDI). SDI is an essential measure of microbial diversity, capturing both the richness and evenness of species present across various time points and treatments. The analysis reveals significant variations in SDI across different time points and treatments. In the time-point analysis, microbial diversity is lowest at Day 0, as indicated by the lower SDI, and gradually increased, reaching its highest level on Day 3. Additionally, the distribution of values broadened over time, suggesting greater variability in microbial composition at later stages (**Figure 2A**). In the treatment analysis, the soil.control exhibited the highest microbial diversity, while the soil.cork treatment showed the lowest SDI, indicating reduced microbial richness (**Figure 2B**). Statistical analysis was carried out using the Kruskal-Wallis test, followed by pairwise comparisons with the Wilcoxon test, resulting in a p-value ≤ 0.05, confirming that these differences in SDI across time points and treatments were statistically significant, thus emphasizing the impact of temporal progression and treatment conditions on the structure of microbial community.

### 3.4 β-diversity indices

**Figure 3:**
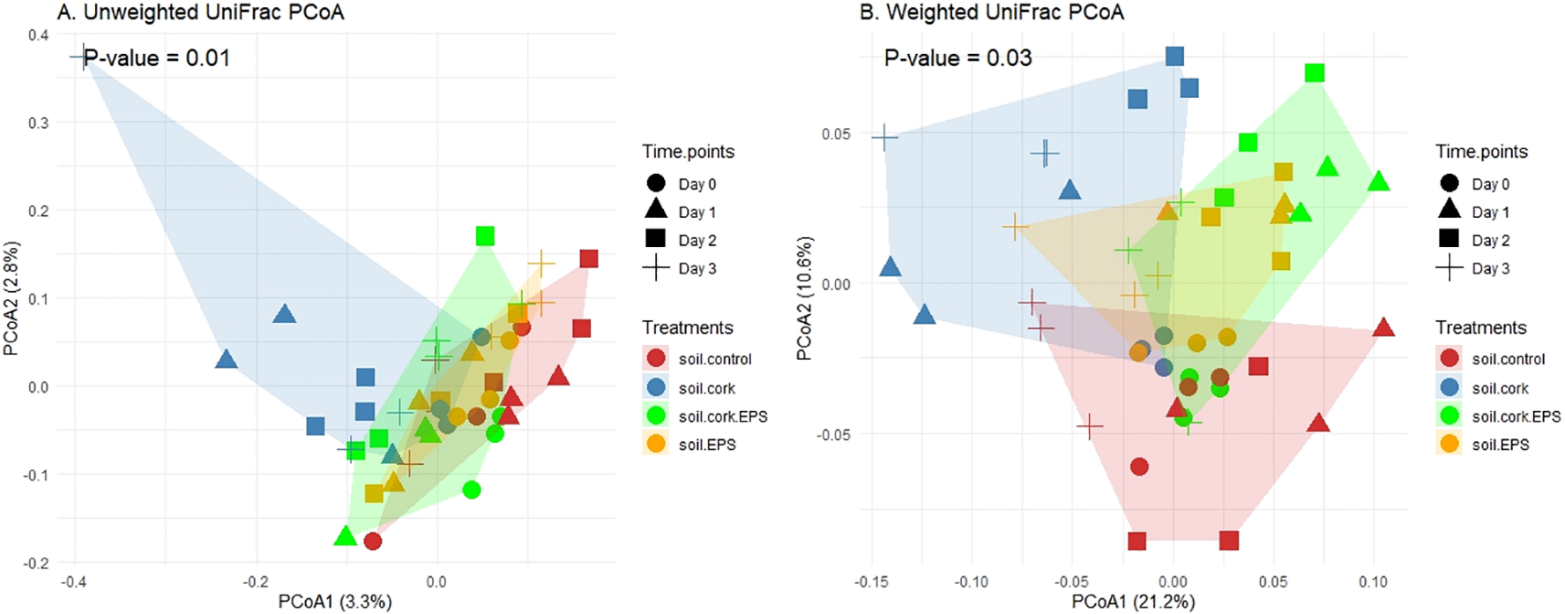
**(A, B)** Beta diversity illustrated through a principal coordinate analysis (PCoA) plot using weighted and unweighted uniFrac measures at various time points and treatments, with statistical significance (p < 0.05). soil.control (soil control), soil.cork (soil+cork), soil.cork.EPS (soil+cork+EPS), soil.EPS (soil+EPS).

The principal coordinate analysis (PCoA) plots illustrate the microbial community differences based on unweighted and weighted UniFrac distances (**Figures 3A, B**). In the unweighted UniFrac PCoA, which considered only the presence or absence of taxa, distinct clustering patterns were observed among time points and treatments, indicating shifts in microbial community composition. Samples from Day 0 were more tightly clustered, whereas later time points, particularly Day 3, exhibited a broader spread, suggesting increased microbial community differentiation over time. Whereas treatments also displayed distinct separation, particularly for soil.cork, highlighting treatment-specific microbial composition (**Figure 3A**).

In the weighted UniFrac PCoA, which accounts for both the relative abundance and taxonomic composition, clearer separation among treatments was observed, particularly along the primary axis (PCoA1), which explains 21.1% of the variation. The soil.control and soil.cork exhibit distinct clustering, indicating significant differences in microbial community structure based on treatment effects. Temporal shifts were also evident, with later time points showing greater dispersion compared to earlier stages. Statistical analysis using PERMANOVA resulted in a p-value of less than 0.05, confirming that the observed clustering patterns in both unweighted and weighted UniFrac analyses were statistically significant. This indicates that both time and treatment were essential in influencing the dynamics of microbial communities (**Figure 3B**).

### 3.5 Venn diagram

**Figure 4:**
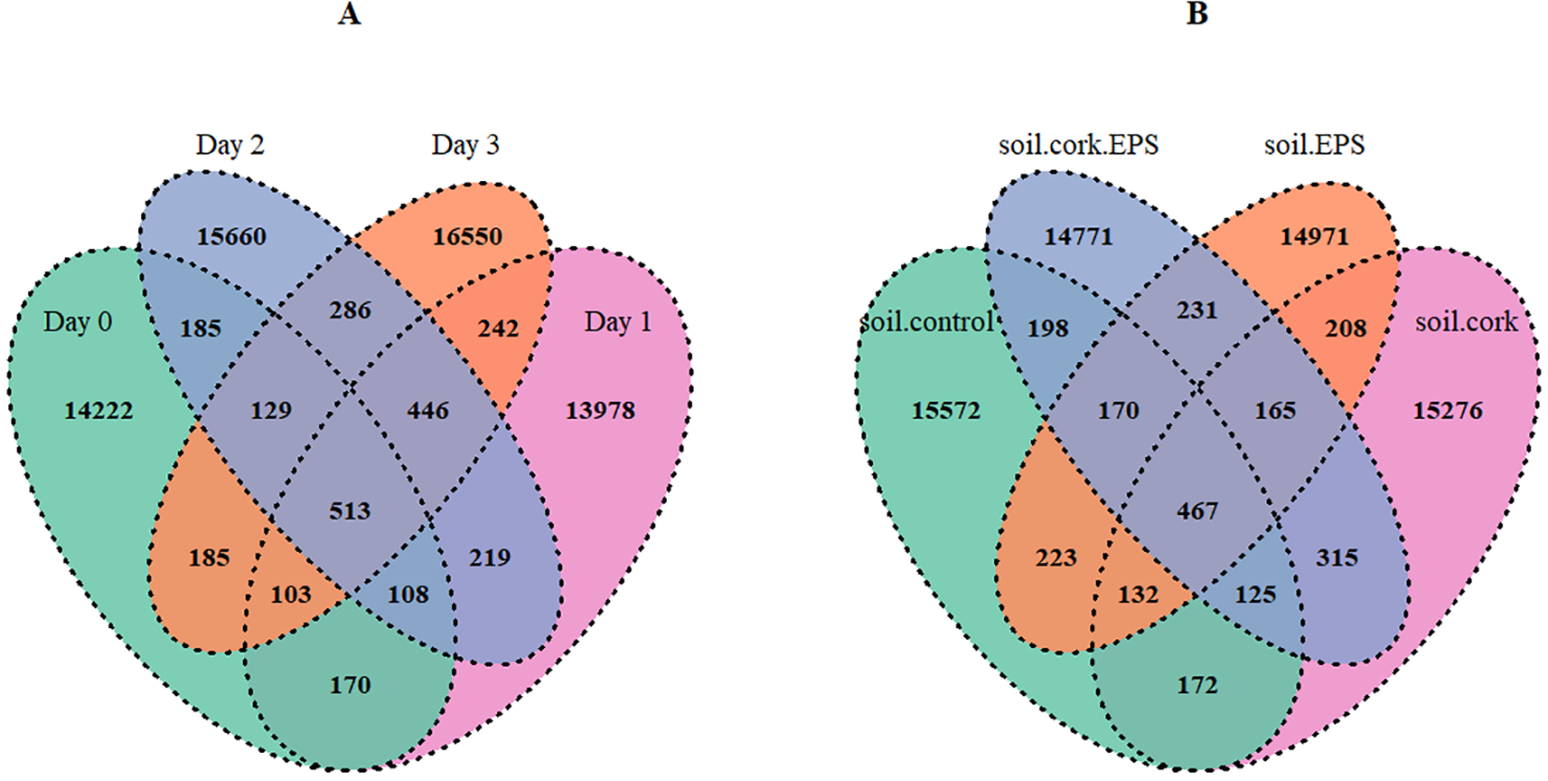
**(A, B)** Venn diagram showing total numbers of shared ASVs across different time points and treatments. soil.control (soil control), soil.cork (soil+cork), soil.cork.EPS (soil+cork+EPS), soil.EPS (soil+EPS).

The Venn diagrams (**Figures 4A, B**) depicted the distribution of ASVs across various time points and treatments, reflecting variations in microbial community composition. The comparison across time points revealed a dynamic shift in microbial diversity, with 14,222 ASVs unique to Day 0, 13,978 to Day 1, 15,660 to Day 2, and 16,550 to Day 3, indicating a progressive expansion of diversity over time (**Figure 4A**). Similarly, differences among soil treatments show that soil.control contained 15,572 ASVs, followed by soil.cork with 15,276, soil.EPS with 14,971, and soil.cork.EPS with 14,771 ASVs (**Figure 4B**). The highest number of unique ASVs in unamended soil implies that microbial diversity was reduced by soil modifications as a result of short-term effects. The overlapping sections in the diagrams represent ASVs shared across different conditions, highlighting core microbiome members that persist despite temporal and treatment-based variations. A total of 62,996 ASVs were identified across all time points and treatments, among which 513 ASVs were shared by all time points, and 467 ASVs were shared across all treatments (**Figures 4A, B**). The presence of both unique and shared ASVs underscores the influence of environmental factors on the dynamics of microbial communities.

### 3.6 Abundance and diversity patterns of microbiota at phylum, class, and family level

**Figure 5:**
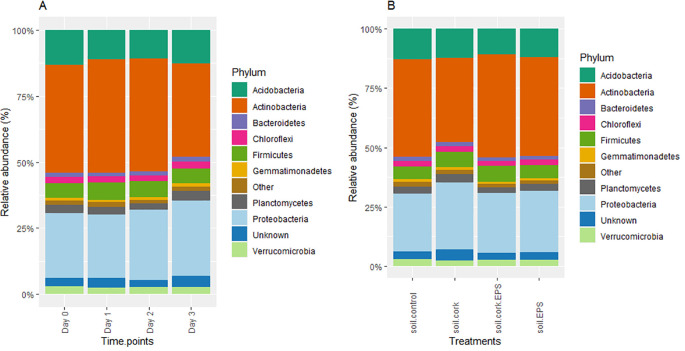
**(A, B)** The relative abundance (%) of bacterial phyla measured across different time points and treatments with statistical significance (*p* < 0.05). soil.control (soil control), soil.cork (soil+cork), soil.cork.EPS (soil+cork+EPS), soil.EPS (soil+EPS).

The predominant groups observed at the phylum level are *Actinobacteria, Proteobacteria*, and *Acidobacteria*, which collectively constitute the majority of the microbial community. The composition remained relatively stable in the time series analysis (Day 0 to Day 3), with *Actinobacteria* consistently being the most prevalent, followed by *Proteobacteria* and *Acidobacteria*. The presence of *Bacteroidetes, Chloroflexi, Firmicutes*, and *Verrucomicrobia* is lower but still contributes to 35% to overall diversity (**Figure 5A**). In the treatment-based comparison (soil.control, soil.cork, soil.cork.EPS, and soil.EPS), similar patterns are observed, with *Actinobacteria* maintaining the highest relative abundance. While minor variations exist among treatments, the microbial community structure remains largely consistent with a p-value of 0.05. Notably, in the soil.cork treatment, *Proteobacteria, Planctomycetes, Firmicutes*, and *Gemmatimonadetes* were found in higher abundance compared to other treatments (**Figure 5B**).

**Figure 6:**
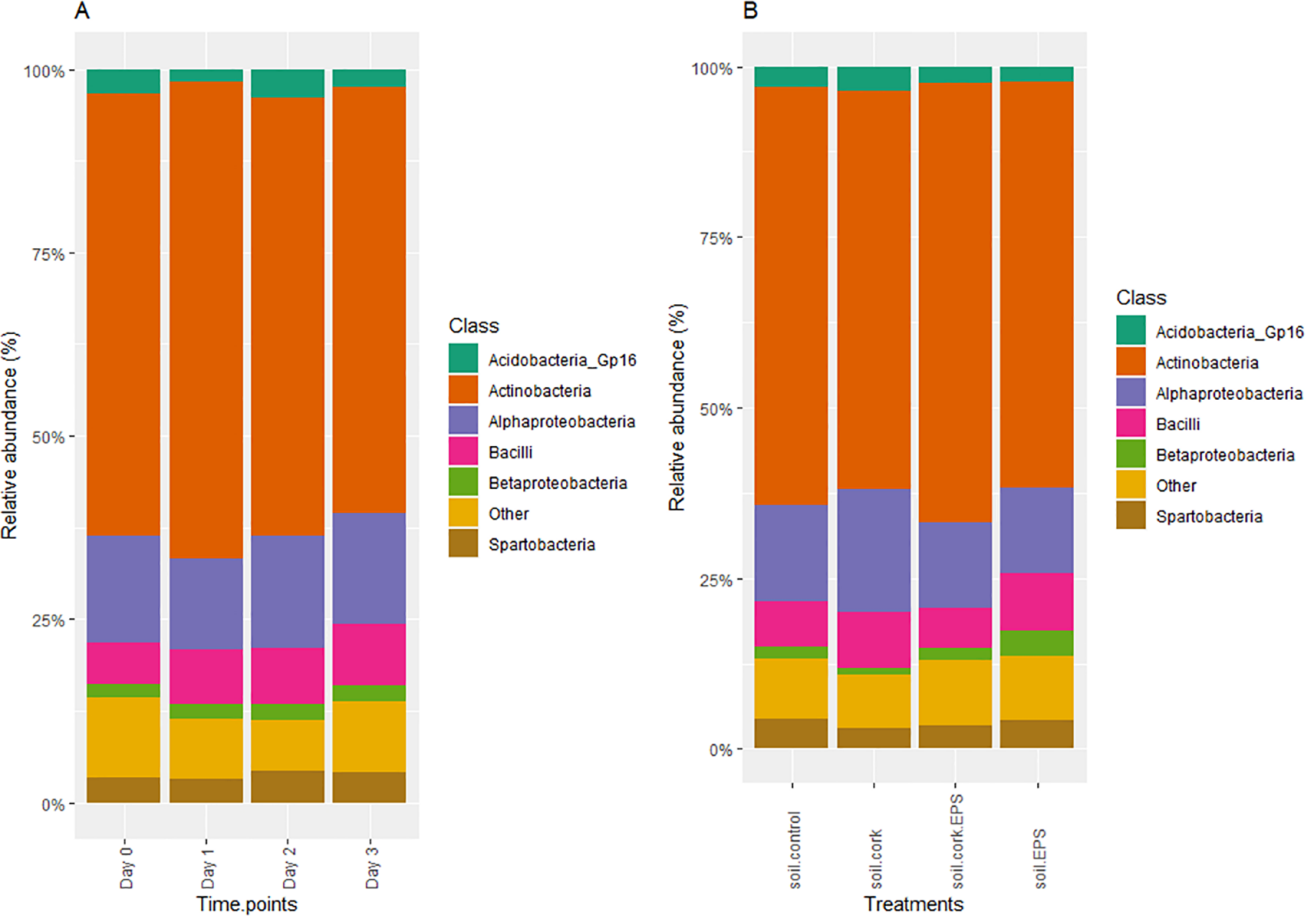
**(A, B)** The relative abundance (%) of bacterial class measured across different time points and treatments with statistical significance (*p* < 0.05). soil.control (soil control), soil.cork (soil+cork), soil.cork.EPS (soil+cork+EPS), soil.EPS (soil+EPS).

At the class level, *Actinobacteria* was shown as the most dominant class, consistently occupying a significant proportion of the microbial community with a p-value of 0.05. Other prominent classes include *Alphaproteobacteria, Bacilli*, and *Betaproteobacteria*, which contribute to microbial diversity in lower proportions. Over time (Day 0 to Day 3), the class composition remained relatively constant, with minor fluctuations in the abundance of *Alphaproteobacteria* and *Bacilli* (**Figure 6A**). Similarly, in the treatment-based analysis (soil.control, soil.cork, soil.cork.EPS, and soil.EPS), the microbial class distribution remains consistent, with *Actinobacteria* continuing to dominate across treatments. The *Acidobacteria_Gp16, Spartobacteria*, and other unclassified classes are present in smaller proportions (**Figure 6B**).

**Figure 7:**
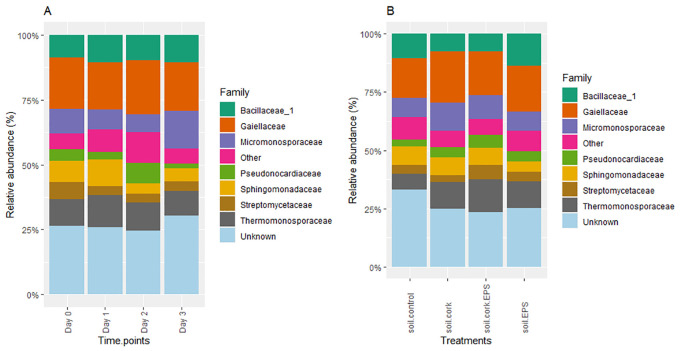
**(A, B)** The relative abundance of bacterial family measured across different time points and treatments with statistical significance (*p* < 0.05). soil.control (soil control), soil.cork (soil+cork), soil.cork.EPS (soil+cork+EPS), soil.EPS (soil+EPS). Unknown indicates that bacteria were either not reported, unknown or unclassified.

At the family level, the relative abundance across different time points and treatments, highlighting key families with a p-value of 0.05 such as *Bacillaceae_1, Gaiellaceae, Micromonosporaceae*, and *Streptomycetaceae*, which dominate the microbial community. Over time (Day 0 to Day 3), the microbial composition remains stable, with *Gaiellaceae* and *Bacillaceae_1* consistently showing high relative abundance, while families like *Pseudonocardiaceae, Sphingomonadaceae*, and *Thermomonosporaceae* contribute to microbial diversity in smaller proportions (**Figure 7A**). Similarly, with the treatments, the core families remain prevalent, with minor variations across treatments. The unknown category constitutes a significant portion, indicating the presence of unclassified taxa. Notably, in the soil.cork, *Thermonosporaceae, Sphingomonadaceae*, and *Micromonosporaceae* exhibited higher relative abundance. Similarly, in the soil.cork.EPS, *Streptomycetaceae* and *Pseudonocardiaceae* were more abundant compared to other treatments. (**Figure 7B**).

### 3.7 Core microbiome analysis

**Figure 8:**
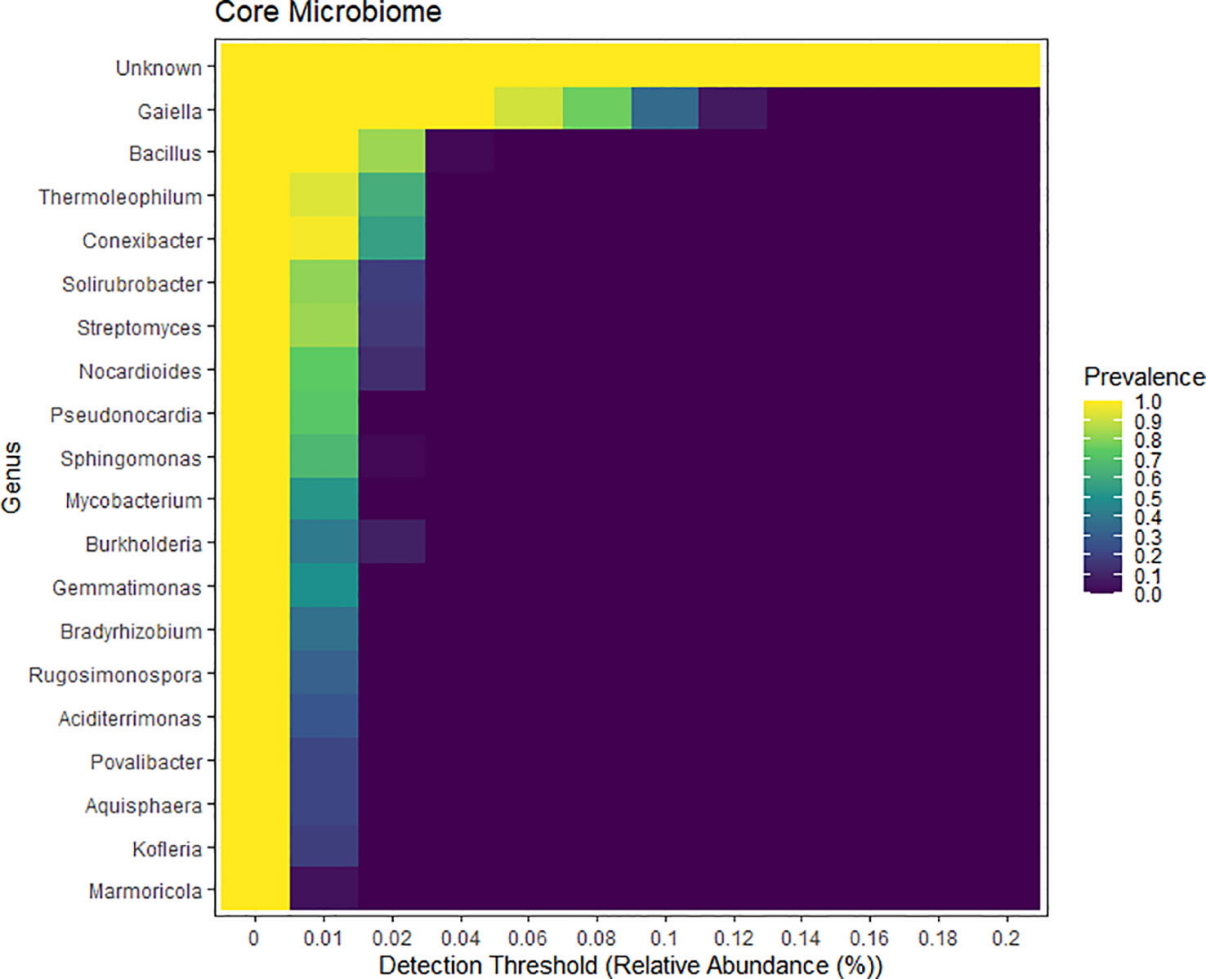
Determination of the top 20 core bacterial microbiome for all treatments. Heatmap of relative abundance for each genus is illustrated on the y-axis. The x-axis depicts prevalence of each relative abundance.

The core microbiome heatmap illustrated the prevalence of various bacterial genera across samples, measured by detection thresholds based on relative abundance. Highly prevalent genera, such as *Bacillus*, *Gaiella*, and an unidentified taxon, appear consistently across all samples with the highest prevalence (ranging from 0.9 to 1.0, indicated in yellow). Other genera, including *Thermoleophilum*, *Conexibacter*, *Streptomyces*, and *Solirubrobacter*, exhibit moderate prevalence, ranging from 0.4 to 0.8. In contrast, taxa such as *Burkholderia*, *Bradyrhizobium*, and *Aquisphaera* are detected at lower frequencies, with prevalence values between 0.1 and 0.3 (depicted in darker shades). The gradient in prevalence highlights differences in microbial community distribution, suggesting that dominant taxa may play important roles in stabilization of soil ecosystem, nutrient cycling, and microbial interactions (**Figure 8**).

## 4 Discussion

Soil amendments are important in modulating microbial communities, with their effects on specific bacterial species varying according to the amendment type. Positive impacts include enrichments in soil microbial biomass and biodiversity, which support resistance and resilience within microbial communities (Aguilar-Paredes et al., 2023). Certain amendments can improve beneficial bacterial diversity while concurrently mitigating the prevalence of harmful pathogens. For instance, increased carbon and nitrogen retention in soils has been shown to elevate populations of beneficial bacteria and a decrease in bacterial wilt across Southern China (Chen et al., 2020). A study reported that there is a significant change in rhizospheric compounds and a reduction of bacterial wilt disease when the soil is amended with biochar, suggesting the role of microbial activity in promoting plant health (Shuang et al., 2021). In contrast, organic amendments can negatively affect the resilience of soil microorganisms to temperature fluctuations, resulting in carbon and nitrogen fluxes in podzol soils, which also affected microbial DNA concentrations. These findings from the previous studies suggested that the strategic application of organic amendments is important in ameliorating environmental stress factors, which directly influence natural soil ecosystems (Kok et al., 2023). Our findings suggest that various soil amendments can interfere with DNA isolation processes, leading to low-quality DNA that requires purification. Utilizing the FastDNA Spin Kit, we consistently obtained high-quality DNA from 48 samples across diverse time points and treatments, which outperformed other DNA isolation methods. Isolating high-quality DNA is crucial for sequencing and our downstream microbial community and diversity analyses.

The advent of 16S rRNA amplicon sequencing has revolutionized our understanding of microbial communities across diverse ecosystems, shedding light into microbial biogeography and ecological functions (Arboleda-Baena et al., 2024; Pattnaik et al., 2020; Dubey et al., 2020). For thorough microbiome analysis, it is crucial to classify query sequences into taxonomic categories by comparing their similarity to sequences in established reference databases, such as SILVA, RDP, and Greengenes (Westcott and Schloss, 2017). Notably, amplicon-based sequencing has provided deeper insights into global microbial biodiversity (Schloss et al., 2016; Rideout et al., 2014) compared to whole-genome sequencing methodologies. Consequently, we employed 16S rRNA amplicon sequencing in this study to analyze the microbial community composition and diversity across various time points and treatments. The addition of cork has been shown to enhance soil structural stability by improving aggregate stability (Nwamba, D. C. 2020). EPS, a byproduct from *Rhizobium tropici*, facilitates soil particle aggregation, ultimately benefiting plants by retaining moisture and sequestering nutrients (Patwardhan et al., 2023; Nivedita et al., 2021). Our study revealed that the combination of cork and EPS significantly influenced soil bacterial diversity as analyzed through amplicon sequencing, which elucidated interspecies relationships and inter-population interactions are critical for plant growth and development.

Microbial diversity is important for nutrient cycling and soil fertility in agriculture. It facilitates nitrogen fixation and decomposition, thereby increasing nutrient availability for crops. Higher levels of microbial diversity are associated with better soil health, including improved respiration and enzymatic activity. Understanding this link is essential for good soil management and sustainable farming. Our study showed that bacterial diversity profiles changed significantly based on the time of sampling and the type of treatment. We measured these changes using the Shannon Diversity Index (SDI) (**Figures 2A** and **2B**). Notably, microbial diversity was lowest on Day 0, as indicated by reduced SDI values, but exhibited a gradual increase, showing its maximum on Day 3 (**Figure 2A**). In treatment comparisons, soil.control demonstrated the highest microbial diversity, whereas soil.cork displayed the lowest SDI values, indicating a reduction in microbial richness (**Figure 2B**). Similarly, the addition of EPS has been shown to influence the biofilm integrity and the composition of microbial communities (Qiu et al., 2021). Recent studies have demonstrated that the application of cattle manure and biochar significantly improved bacterial alpha-diversity profiles in tea-planting soils, thereby improving the soil structure (Han et al., 2022). The addition of modified biochar to tea-planting soils resulted in an increase in the bacterial community’s SDI, ACE, and Chao1 indices by 3.05%, 5.07%, and 5.24%, respectively (Hua et al., 2021). Further, research revealed unique differences in bacterial alpha-diversity, represented by Shannon and Chao1 indices, among soils from major land use types characterized as highly contaminated (HC), low contaminated (LC), and non-contaminated (NC) (Li et al., 2022).

A comparative study across three different locations (West Sussex, UK; Lusignan, France; and Prato Sesia, Italy) revealed variations in bacterial beta diversity influenced by biochar treatments when compared with controls. Among the locations, UK and Italy, shown significant changes in bacterial composition and abundance, which attribute to changes in edaphic factors, such as soil pH (Jenkins et al., 2017). Our findings similarly indicated distinct temporal patterns in community clustering based on weighted and unweighted UniFrac distances with PERMANOVA analysis (p < 0.05) across different time points and treatment conditions (**Figure 3A, B**). Another study with the PERMANOVA analysis of weighted UniFrac distances revealed significant differences (p < 0.01) in bacterial community composition at varying levels of RN infestation, with PCoA plots illustrating clear clustering of samples sharing similar bacterial profiles, underscoring the impact of RN infestation on the structure of bacterial communities (Karapareddy et al., 2025). In sites across China (Kaihua, KH; Shenzhou, SZ; and Quzhou, QZ), a study on the temporal dynamics of beta diversity among niche soil communities demonstrated noticeable changes, when these sites have been treated with biogas slurry over three years sequentially and compared against controls (Xing et al., 2023).

Previous studies comparing the compositional and functional profiles of bacterial communities treated with compost (C+) and without compost (C-) soils using 16S rRNA sequencing revealed that *Proteobacteria* were predominant in both treatments, followed by *Chloroflexi, Actinobacteria, Verrucomicrobia, Firmicutes*, and *Bacteroidetes*. Specifically, *Firmicutes* were dominant in C+ soils, while *Chloroflexi* exhibited greater prevalence in C- soils (Kim et al., 2021). In this study, our time point analysis (Day 0 to Day 3) demonstrated stability in bacterial composition, with *Actinobacteria* consistently being the most abundant, followed by *Proteobacteria* and *Acidobacteria* (**Figure 5A**). Similarly, our treatment-based comparisons (soil.control, soil.cork, soil.cork.EPS, and soil.EPS) identified *Actinobacteria* as a dominant phylum (**Figure 5B**). At the class level, from Day 0 to Day 3, composition remained largely stable, with slight fluctuations in the relative abundance of *Alphaproteobacteria* and *Bacilli* (**Figure 6A**). Treatment-based analysis indicated consistent microbial class distributions, with *Actinobacteria* predominating across all treatments (**Figure 6B**). Similarly, another study identified *Proteobacteria* as the dominant phylum, followed by *Actinobacteria* and *Bacteroidetes* when the soils have been amended with Metarhizium (fungus) (Barelli et al., 2020). In watermelon cultivation, the use of composted cattle and chicken manure, combined with bioorganic fertilizers, effectively suppressed Fusarium wilt. Whereas, Proteobacteria*, Actinobacteria, Chloroflexi*, and *Planctomycetes* have been determined as dominant phyla, which is similar to findings identified in this study. Further they suggested that bio-organically treated soils may have promoted a few novel microbial communities that modified the rhizosphere composition of watermelon (Ayangbenro et al., 2022).

Furthermore, another study indicated that soil amendments, including bio-organic fertilizers and compost from cow and chicken manure, significantly altered the bacterial community structure compared to untreated control soils, subsequently enhancing watermelon quality and reducing Fusarium incidence (Zhao et al., 2018). In our investigation, results at the family level showed a consistent relative abundance across various time points and treatments, highlighting dominant taxa such as *Bacillaceae_1, Gaiellaceae, Micromonosporaceae*, and *Streptomycetaceae*. The microbial composition exhibited stability over time, as depicted in **Figure 7A**. Additionally, the treatments maintained a generally consistent order of core families in terms of abundance, with minor variations noted (**Figure 7B**). Another study identified profound shifts in bacterial ecology when the soil was amended with varying rates of sewage sludge (0: CK; 30: ST; 75: MT; and 150: HT t/ha) on a dry weight basis. The dominant families identified include *Xanthomonadaceae, Hyphomicrobiaceae*, and *Flavobacteriaceae*, with average relative abundances of 36.2%, 9.6%, and 3.8%, respectively (Li et al., 2021).

In our findings, the top 20 core microbiomes consistently featured were *Bacillus* and *Gaiella* across all treatments, with their prevalence ranging from 0.9 to 1.0. Other genera such as *Thermoleophilum, Conexibacter, Streptomyces*, and *Solirubrobacter* exhibited moderate prevalence (0.4 to 0.8), while taxa like *Burkholderia, Bradyrhizobium*, and *Aquisphaera* were less prevalent (0.1 to 0.3) (**Figure 8**). Furthermore, another study reported core microbiomes across three planting systems (conventional, aerobic, and System of Rice Intensification, SRI) in saline environments identified 43 genera, including *Sphingomonas, Sorangium, Nitrospira, Luteitalea, Candidatus_Solibacter*, and *Bacillus*, which were found to have higher prevalence (Rokins et al., 2022). Similarly, research focusing on chemical and organic fertilizers revealed twelve predominant core bacterial genera: *Acidobacterium, Bacillus, Bradyrhizobium, Clostridium, Gemmatimonas, Lysobacter, Massilia, Pseudomonas, Rhizobium, Sphingomonas, Stenotrophomonas*, and *Xanthomonas*. Of which, *Bacillus* showed the highest relative abundance in soils supplemented with neem cake, contrasting with the lowest levels found in soils amended with castor cake (Su et al., 2022). Additional findings demonstrated a strong positive correlation between the relative abundances of *Nocardioides, Ilumatobacter*, and *Gaiella* under organic amendment (vermicompost), which effectively inhibited *Fusarium oxysporum* f. sp. *lycopersici* during tomato cultivation, compared to rice straw and chicken manure treatments (Zhao et al., 2019).

Certain bacterial genera identified in our study play a critical role in maintaining the health of both plants and soil. For example, Bacillus species aid in nitrogen cycling and breaking down organic matter, which boosts soil fertility and helps plants grow (Nishisaka et al., 2024). Similarly, *Gaiella* has been implicated in carbon and nitrogen cycling, increasing nutrient availability when treated with long-term organic and inorganic fertilization (Li et al., 2017). *Streptomyces* play a vital role in organic matter breakdown, contributing to carbon and nitrogen cycling; when introduced to soils with organic amendments like compost, they improve nutrient uptake and support the growth of crops such as cabbage (*Brassica oleracea*) and tomato (Hu et al., 2021). *Burkholderia* species are crucial for nitrogen fixation, with their inoculation, along with compost or biochar, shown to enhance nitrogen availability and yields in soybean (*Glycine max*) (Meier et al., 2021). *Thermoleophilum* and *Solirubrobacter* are thermophilic genera that contributes to organic matter decomposition (Schroder et al., 2021; Han et al., 2024), significantly impacting nutrient cycling, especially within thermophilic composting systems, thereby improving soil health and fertility in crops like lettuce (*Lactuca sativa*).

Furthermore, to understand how microbial diversity changes and its role in ecosystem functioning, it is essential to assess soil health. Key indicators used to evaluate microbial diversity and its ecological roles include microbial biomass, phospholipid fatty acid analysis (PLFA), enzyme activity, organic carbon levels, and nitrogen cycling. Microbial biomass indicates microbial presence and ecosystem resilience, whereas PLFA reveals the composition of microbial communities, which is essential for efficient nutrient cycling. Soil enzyme activities indicate functional diversity, while organic carbon content serves as an indicator for potential carbon sequestration, which is closely linked to microbial diversity. It is also essential to understand the composition and diversity of microbes in nitrogen cycling in order to maximize plant productivity and improve water retention. In this study, we determined microbial composition and diversity using amplicon sequencing and analysis. The cork and EPS amendments together have been demonstrated to aid in improving soil health and increasing agricultural productivity (**Supplementary Table 3**). Synergistically, these amendments improve soil structure, porosity, and aeration, which contributes to improved water retention and increased nutrient availability. These edaphic changes promote plant growth and facilitate the formation of a rich and diverse microbial community, which is crucial for nutrient cycling. In addition, these amendments can sequester carbon, reduce greenhouse gas emissions, control soil erosion, and lower irrigation needs, particularly in arid and acidic soils. Collectively, these practices contribute to improved soil quality and increased crop yields, thereby supporting sustainable agricultural systems.

## 5 Conclusions

Soil microorganisms play a crucial role in biomass production, nutrient cycling, and the decomposition of organic matter. Biopolymer amendments, cork and EPS, enhance soil health by improving its structure and moisture retention. Together, they support microbial communities and resilient ecosystems. Microbial community characterization and the pursuit of rhizosphere ecology are primarily being pursued by next-generation sequencing (NGS) and microbiome investigations. Understanding the impact of organic additives on DNA isolation and microbial composition is imperative. Therefore, this study validated six DNA extraction protocols as a prelude to utilizing NGS to dissect soil microbial composition. After testing these methods on unamended and amended soils, the results show that the FastDNA Spin Kit is the most effective for extracting DNA from diverse microbial populations. Amplicon sequencing of the 16S rRNA gene revealed distinct shifts in the microbial community over time and across treatments. Both α-diversity and β-diversity analyses confirmed significant changes in microbial diversity over time and across treatments. Venn diagram analysis further highlighted the presence of unique, common, and shared ASVs across treatments and time points, emphasizing the dynamic nature of soil microbial communities. In total, this study identified twelve genera through core microbiome analysis across various time points and treatments, highlighting their crucial roles in maintaining soil ecosystem stability, carbon sequestration, nitrogen fixation, sulfur cycling, and phosphorus solubilization. In summary, this study provides valuable insights into the microbial dynamics of amended soils. It first highlights the need for careful selection of DNA extraction methods and then discusses the steps involved in amplicon sequencing followed by phyloseq analysis for reliable microbial profiling. These findings help us understand how soil microbes respond to biopolymers, which can lead to improved sustainable soil management and agriculture.

## Data Availability

The datasets presented in this study can be found in online repositories. The names of the repository/repositories and accession number(s) can be found below: https://www.ncbi.nlm.nih.gov/, SAMN47267746 - SAMN47267793.
