# Supplementary material for: Effects of biopolymers, cork, and Rhizobium tropici-derived extracellular polymeric substances on soil microbial communities
Source: Front Microbiomes. 2025 Aug 15;4:1614472. doi: 10.3389/frmbi.2025.1614472 (PMC12993683; doi:10.3389/frmbi.2025.1614472)
Supplement: Supplementary file 1 [file Table1.docx]

**Supplementary Table 1.** **Chemical Properties of Decatur Soils (Textural Analysis)**

| Sample ID | Sand (%) | Silt /Clay (%) | Textural Class | Nitrogen (%) | Carbon (%) | Sulfur (%) |
| --- | --- | --- | --- | --- | --- | --- |
| Dsl1 | 26.31 | 69.45 | Silt Loam | 0.22 | 8.45 | 0.19 |
| Dsl2 | 17.50 | 78.00 | Silt Loam | 0.21 | 8.51 | 0.18 |
| Dsl3 | 21.91 | 73.73 | Silt Loam | 0.22 | 8.48 | 0.19 |

**Footnote:** Dsl1-3, Decatur Silt Loam, 1-3.
