# Supplementary material for: Effects of biopolymers, cork, and Rhizobium tropici-derived extracellular polymeric substances on soil microbial communities
Source: Front Microbiomes. 2025 Aug 15;4:1614472. doi: 10.3389/frmbi.2025.1614472 (PMC12993683; doi:10.3389/frmbi.2025.1614472)
Supplement: Supplementary file 2 [file Table2.docx]

**Supplementary Table 2.** Sequence read summary table of the unamended and amended soils categorized on day-wise treatment.

| Sample No. | Day_wise_Treatments | Raw_seqs (R1+R2) | Trimmed_seqs (R1+R2) | Chimera_free_seqs | Seqs (after_size_filtration) | Final_unique_seqs |
| --- | --- | --- | --- | --- | --- | --- |
| 1 | SOIL.CONTROL.DAY.0 | 658558 | 653072 | 274173 | 240328 | 6091 |
| 2 | SOIL.CORK.DAY.0 | 655398 | 650746 | 262592 | 229608 | 5800 |
| 3 | SOIL.EPS.DAY.0 | 727676 | 722090 | 298316 | 262909 | 6396 |
| 4 | SOIL.CORK.EPS.DAY.0 | 728694 | 722660 | 303334 | 265934 | 6511 |
| 5 | SOIL.CONTROL.DAY.1 | 758870 | 752972 | 311764 | 278219 | 6230 |
| 6 | SOIL.CORK.DAY.1 | 833964 | 826940 | 381446 | 371286 | 5627 |
| 7 | SOIL.EPS.DAY.1 | 612412 | 607958 | 253828 | 224875 | 5174 |
| 8 | SOIL.CORK.EPS.DAY.1 | 680628 | 675274 | 279046 | 252200 | 4975 |
| 9 | SOIL.CONTROL.DAY.2 | 826180 | 820226 | 337700 | 299825 | 7026 |
| 10 | SOIL.CORK.DAY.2 | 752870 | 747176 | 311925 | 281781 | 5623 |
| 11 | SOIL.EPS.DAY.2 | 751904 | 745904 | 306508 | 273118 | 5876 |
| 12 | SOIL.CORK.EPS.DAY.2 | 837654 | 831228 | 341264 | 310643 | 5759 |
| 13 | SOIL.CONTROL.DAY.3 | 743502 | 738148 | 322073 | 298277 | 6649 |
| 14 | SOIL.CORK.DAY.3 | 949300 | 941292 | 426748 | 402060 | 5064 |
| 15 | SOIL.EPS.DAY.3 | 917688 | 910168 | 383207 | 343905 | 7467 |
| 16 | SOIL.CORK.EPS.DAY.3 | 910228 | 902398 | 384080 | 348071 | 7089 |

Footnote: Soil.Control: Soil Control, Soil.Cork: Soil and Cork, Soil.EPS: Soil and EPS, and Soil.Cork.EPS: Soil and Cork and EPS.
