# Supplementary material for: Effects of biopolymers, cork, and Rhizobium tropici-derived extracellular polymeric substances on soil microbial communities
Source: Front Microbiomes. 2025 Aug 15;4:1614472. doi: 10.3389/frmbi.2025.1614472 (PMC12993683; doi:10.3389/frmbi.2025.1614472)
Supplement: Supplementary file 3 [file Table3.docx]

**Supplementary Table 3.** **Potential functional roles of significant microbes found in amended (cork and EPS) soils.**

| Species | Potential Functional Roles |
| --- | --- |
| *Sphingomonas spp.* | Promote plant germination and growth, degrade organic pollutants, including polycyclic aromatic hydrocarbons, and remediate environmental contaminants to produce highly beneficial phytohormones. |
| *Ralstonia picketti* | It can degrade biaryl compounds in soil, with rates affected by environmental factors. |
| *Psuedoduganella eburnea* | Carbon cycling. It produces enzymes such as reductases, which are responsible for the biosynthesis of nanoparticles. |
| *Phenylobacterium kunshanese* | It is known for its potential in bioremediation and degradation of organic compounds. |
| *Herbaspinilllum huttliiense* | Nitrogen-fixing bacteria. It can be found in association with plant roots and may be involved in promoting plant growth. |
| *Phyllobacterium brassicacearum* | Plant growth-promoting rhizobacterium. It can enhance water-use efficiency in plants, making them more resilient to drought conditions. |
| *Paraburkholderia spp,* | Soil priming, carbon cycling, and prevent soil-borne diseases. It can influence plant health and nutrient availability. |
| *Neobacillus Cucumis* | It can improve soil properties, such as organic matter content, soil structure, and water retention. |
| *Neobacillus spp.* | It contributes to crucial soil ecological functions, promotes plant growth, and enhances stress tolerance in plants. |
| *Neobacillus dielmonensis* | It plays a crucial role in improving soil quality and promotes plant growth. |
| *Neobacillus niacin* | It can promote plant growth and potentially aid in soil remediation. |
